# Neuroprotection by Phytoestrogens in the Model of Deprivation and Resupply of Oxygen and Glucose In Vitro: The Contribution of Autophagy and Related Signaling Mechanisms

**DOI:** 10.3390/antiox9060545

**Published:** 2020-06-22

**Authors:** Giuseppe Abbruzzese, Javier Morón-Oset, Sabela Díaz-Castroverde, Nuria García-Font, Cesáreo Roncero, Francisco López-Muñoz, José Luis Marco Contelles, María Jesús Oset-Gasque

**Affiliations:** 1 Department of Biochemistry and Molecular Biology, Faculty of Pharmacy, Complutense University of Madrid, E-28040 Madrid, Spain; giu.abbru@gmail.com (G.A.); javimoroset@gmail.com (J.M.-O.); sabelacastroverde@gmail.com (S.D.-C.); nuriagarciafont@ucm.es (N.G.-F.); ceronce@ucm.es (C.R.); 2 Health Research Institute of the Hospital Clínico San Carlos (IdISSC), 28040 Madrid, Spain; 3 Faculty of Health, Camilo José Cela University of Madrid (UCJC), 28692 Madrid, Spain; flopez@ucjc.edu; 4 Neuropsychopharmacology Unit, “Hospital 12 de Octubre” Research Institute, 28041 Madrid, Spain; 5 Laboratory of Medicinal Chemistry, Institute of Organic Chemistry (CSIC), Juan de la Cierva 3, 28006 Madrid, Spain; jlmarco@iqog.csic.es; 6 Instituto de Investigación en Neuroquímica, Universidad Complutense de Madrid, Ciudad Universitaria, 28040 Madrid, Spain

**Keywords:** apoptosis, autophagy, brain ischemia, cell signaling, natural antioxidants, neuroprotection, phytoestrogens

## Abstract

Phytoestrogens can have a neuroprotective effect towards ischemia-reperfusion-induced neuronal damage. However, their mechanism of action has not been well described. In this work, we investigate the type of neuronal cell death induced by oxygen and glucose deprivation (OGD) and resupply (OGDR) and pinpoint some of the signaling mechanisms whereby the neuroprotective effects of phytoestrogens occur in these conditions. First, we found that autophagy initiation affords neuronal protection upon neuronal damage induced by OGD and OGDR. The mammalian target of rapamycin/ribosomal S6 kinase (mTOR/S6K) pathway is blocked in these conditions, and we provide evidence that this is mediated by modulation of both the 5′ AMP-activated protein kinase (AMPK) and phosphatidylinositol-3-kinase/protein kinase B (PI3K/AKT) pathways. These are dampened up or down, respectively, under OGDR-induced neuronal damage. In contrast, the MAPK-Erk kinase/extracellular signal-regulated kinase (MEK/ERK) pathway is increased under these conditions. Regarding the pathways affected by phytoestrogens, we show that their protective properties require autophagy initiation, but at later stages, they decrease mitogen-activated protein kinase (MAPK) and AMPK activation and increase mTOR/S6K activation. Collectively, our results put forward a novel mode of action where phytoestrogens play a dual role in the regulation of autophagy by acting as autophagy initiation enhancers when autophagy is a neuroprotective and pro-survival mechanism, and as autophagy initiation inhibitors when autophagy is a pro-death mechanism. Finally, our results support the therapeutic potential of phytoestrogens in brain ischemia by modulating autophagy.

## 1. Introduction

Ischemic stroke is the third cause of death worldwide [1]. In this condition, oxygen and glucose supply to the brain is impaired, causing metabolic distress, which may lead to glutamate-mediated excitotoxicity [2] and cell death in the core of the lesion, whereas the penumbra becomes functionally silent but remains structurally intact [3]. Three ways of cellular death have been implicated in stroke: necrosis, apoptosis, and, most recently, autophagy. Necrosis predominates in the ischemic core, whereas apoptosis and autophagy are often observed in the penumbra after focal ischemia [3]. The relationship between these three types of death in ischemia is not clear. Despite improvements in stroke management over the last decade, mainly due to the use of thrombolysis, there is no effective neuroprotective treatment against the acute phase of stroke [1,4].

Autophagy has emerged in recent years as an essential mechanism for maintaining cellular homeostasis and as part of the cellular response to stress [5,6]. However, numerous reports have documented that both ischemia- and reperfusion-induced excessive autophagy activation leads to neuronal death through unknown mechanisms [7,8]. Nonetheless, autophagy can be protective during post-stroke recovery by diminishing the degeneration of disrupted neurites, clearing damaged mitochondria, and stopping apoptosis and necrosis [9].

The increasing knowledge about the ischemic cascade has led to a considerable development of pharmacological tools, suggesting that each step of this cascade might be a target for cytoprotection. Thus, many neuroprotective drugs such as calcium channel blockers [10], antioxidants or free radical scavengers [11,12], gamma-aminobutyric acid (GABA) agonists, glutamate antagonists, NO inhibitors, growth factors, anti-inflammatories, and phosphatidylcholine precursors as citicoline [12,13] have been studied in experimental stroke models and stroke patients. However, very few have shown efficacy in clinical trials. Among them, caspase inhibitors [14] to reduce apoptosis and estrogens or its derivatives, phytoestrogens [15,16], have been proposed as future neuroprotective agents.

In vivo and in vitro studies have demonstrated the powerful neuroprotective effects of estrogens against a variety of ischemic insults [16,17,18], which appear to use multiple mechanisms depending on the injury.

Interestingly, it is increasingly clear that physiological doses of isoflavones can mimic some of the neuroprotective effects of estrogens [19,20]. Phytoestrogens are a group of plant-derived compounds that mainly comprise isoflavones. It has been shown that phytoestrogens prevent neuronal damage and improve the outcome under experimental stroke conditions [21,22,23]. Emerging findings suggest that phytoestrogens have the ability to target multiple pathophysiological processes involved in stroke, including oxidative stress, inflammation, and apoptotic cell death [24,25,26,27]. Furthermore, epidemiological studies suggest that the consumption of plant sources rich in phytochemicals may reduce stroke risk, which reinforces the possibility of developing preventive or neuroprotective therapies for stroke [28]. However, the mechanism of action whereby these compounds exert their neuroprotective effects is poorly understood. Importantly, in the majority of published works, estrogens and phytoestrogens were studied as neuroprotective agents, but their possible effects on neurorepair in the post-ischemic period have not been described, and it is at this point where protection is necessary after an ischemic event.

Recent results reported by Oset-Gasque et al. demonstrated that the phytoestrogen Genistein may protect neurons against oxygen and glucose deprivation (OGD) and resupply (OGDR) by attenuating oxidative stress, lipid peroxidation, and necrotic cell death [29]. Moreover, genistein can also reduce necrosis and increase neurons viability by modulating autophagy [29]. They also observed a decrease in reactive species of oxygen (ROS) due to a regulation of hypoxia inducible factor α (HIFα), which could control the activation of autophagic proteins such as Beclin1 and Bcl2 and adenovirus E1B 19 kDa-interacting protein 3 (BNIP3). Recent results have also demonstrated that the phytoestrogen Daidzein has cytoprotective properties in neurons, which are due to an increase in peroxisome proliferator-activated receptor gamma (PPARγ) activity [30]. The phytoestrogen Genistein exhibited neuroprotective effects following ischemic stroke in rats by targeting NF-κB signaling and possibly by inhibiting downstream inflammatory immune cells in the brain [31]. Similarly, the phytoestrogen biochanin A provided neuroprotection against cerebral ischemia-reperfusion injury by Nrf2-mediated inhibition of oxidative stress and inflammation signaling pathway in rats [32]. All in all, these results indicate that genistein, biochanin A, and daidzein could have a therapeutic potential in neuroprotection and neurorepair.

The present study focuses on the potential neuroprotective effects and the mechanism of action of phytoestrogens genistein and biochanin A, compared to estrogens (17β-estradiol). To this end, neuronal primary cultures were subjected to OGD and OGDR to investigate the involvement of autophagy and the signaling pathways of mitogen-activated protein kinase (MAPK), phosphatidylinositol-3-kinase/protein kinase B (PI3K/AKT), and 5′ AMP-activated protein kinase/ mammalian target of rapamycin/p70 ribosomal S6 kinase (AMPK/mTOR/p70S6K) as potential mediators of the neuroprotective effects exerted by phytoestrogens against this type of damage.

## 2. Materials and Methods

### 2.1. Primary Cortical Neuron Cultures

Cortical neurons were obtained from fetal Wistar rat brains at gestation day 19, following the procedure described in [33], with minor modifications. In these experiments, all animal handling was performed in accordance with European Commission guidelines (2010/63/UE) and was approved by the Animal Research Committee at the Complutense University (ethical code: PROEX012/18). Briefly, after eliminating meninges and blood vessels, the brain cortex was homogenized in HBSS medium (Hanks Buffer Salt Solution 1X, Gibco, Thermo Fisher Scientific, Madrid, Spain), and filtered in a falcon tube with a filter (54 μm). The tissue homogenate was washed by centrifugation twice with a Krebs-HEPES solution (Locke’s calcium-free medium) containing (in mM) NaCl 140, KCl 4.7, KH_2_PO_4_ 1.2, glucose 5.5, ascorbic acid 0.5, EGTA 1 and HEPES 10, pH 7, and once with Dulbecco’s Modified Eagle’s Medium (DMEM) F12-Glutamax (Gibco, Thermo Fisher Scientific, Madrid, Spain). After counting, cells were seeded in DMEM F12-Glutamax containing 10% fetal bovine serum (FCS) and 1% of a mix of antibiotics and antimycotics (Gibco^®^ Antibiotic-Antimycotic, Thermo Fisher Scientific, Madrid, Spain). This solution containing 10,000 units/mL of penicillin, 10,000 µg/mL of streptomycin, and 25 µg/mL of Fungizone^®^. Cells were then plated in poly-D-lysine (0.04 mg/mL, Sigma Aldrich, Madrid, Spain)-coated plates and then incubated in a 5% CO_2_ atmosphere at 37 °C. After 3–4 d, the medium was changed, and 1% B27 supplement (Gibco, Thermo Fisher Scientific. Madrid, Spain) was added to the culture medium instead of FCS. Cells were seeded at different densities depending on the type of experiment performed.

### 2.2. Oxygen and Glucose Deprivation (OGD) and Resupply (OGDR)

For this study, we used an in vitro OGD and OGDR model, where cultured cells were subjected to short-term oxygen and glucose deprivation, followed by subsequent resupply of these. Ischemia represents the interruption of metabolic fuel and oxygen delivery to support cellular oxidative metabolism, and reintroduction of oxygen upon reperfusion of ischemic tissue triggers oxidative stress, which initiates the reperfusion injury cascade culminating in injury and death of cells and tissues [34]. Thus, cultured cells subjected to hypoxia, fuel deprivation, and reoxygenation replicate the cardinal features of ischemia–reperfusion. This is a commonly used model to investigate changes in gene and protein expression associated with this type of insult, as well as to test the neuroprotective properties of drugs and their molecular effects on signaling pathways. These detailed studies are not as feasible using more complex systems, especially intact animals [34].

Thus, firstly, cells were subjected to OGD [29]. The DMEM F12 Glutamax was replaced with DMEM glucose-free medium and transferred to an anaerobic incubator in a 95% N_2_/5% CO_2_ atmosphere at 37 °C for 3 h. At the end of this period, the cells were treated for 6–24 h, as indicated, with drugs. For this purpose, the glucose-free medium was replaced with the DMEM F12 Glutamax, a medium containing glucose with the mixture of antibiotics/antimycotics (1%) and B27 supplement (1%; culture medium) and cells were kept in a normoxic incubator (95% Air/5% CO_2_ atmosphere at 37 °C). The treatment with phytoestrogens was always done after the OGD period, that is, during the OGDR treatment. The compounds used were genistein (Gen), biochanin A (BioA), and 17-β estradiol (BE) (all of them from Sigma Aldrich, Madrid, Spain) at concentrations between 0.001–100 µM, as indicated in each experiment. For autophagy inhibition, 5 mM 3-methyladenine (3-MA; Sigma Aldrich, Madrid, Spain) was used. Inhibitors of cellular pathways LY294002 (AKT inhibitor) and PD98059 (MEK inhibitor) (Calbiochem, Sigma Aldrich, Madrid, Spain), at 1μM, were used. Control plates were kept in the normoxic incubator (95% Air/5% CO_2_ atmosphere at 37 °C) for the same time period of the OGD and then replaced with the DMEM F12-Glutamax fresh medium.

### 2.3. Western Blotting

For these experiments, cells were seeded in 5 cm Petri dishes Falcon^®^ (CULTEK, S.L.U., Madrid, Spain) at a density of 10^6^ cells/mL.

Cell extracts were prepared using a lysis buffer containing 1% Triton, 10 mM Tris, 5 mM EDTA, 50 mM NaCl, 30 mM sodium pyrophosphate, 50 mM NaF, 100 µM orthovanadate, 500 µM phenylmethylsulfonyl fluoride, 5 µg/L aprotinin, and 0.025 µg/L leupeptin, pH 7.4. After scraping the plates, lysates were used to measure proteins concentration with the Bradford method. The samples were heated for 10 min at 95 °C and then separated with electrophoresis in 10% polyacrylamide gels. Subsequently, the proteins were transferred to a nitrocellulose membrane. After blocking the non-specific sites with TBST (Tris-Buffered Saline, 0.1% Tween^®^ 20 Detergent) and milk 5% for 1 h, the membranes were incubated with primary antibodies. The immunodetection was made by enhanced chemiluminescence (ECL) of luminol, after incubating the secondary antibodies. All the antibodies used for these experiments, as well as the used dilutions, are indicated in Table 1A.

### 2.4. Immunocytochemistry

Cells were seeded in 24-well plates on glass coverslips and treated with phytoestrogens at 1 μM to observe the expression of microtubule-associated protein light chain 3B (LC3B) and βIII Tubulin (B3T). Every treatment was performed in the absence or presence of 5 mM 3-MA. After 24 h, the medium was removed, cells were washed with phosphate-buffered saline (PBS) and fixed with 4% paraformaldehyde in PBS for 15 min. Coverslips were stored at −80 °C in solution cryoprotectant (30% ethylene glycol, 30% glycerol, 40% 0.1M phosphate buffer). Cells were washed again with PBS before immunodetection. The following antibodies were used (Table 1B). Coverslips were washed three times with PBS and then mounted on glass slides with Prolong Antifade from Invitrogen (Thermo Fisher Scientific, Madrid, Spain), a reagent that suppresses photobleaching and preserves the signals of fluorescently labeled target molecules for long-term storage and analysis. Preparations were allowed to dry for at least 24 h before performing detailed observation under the confocal microscope. LC3B fluorescence was quantified by fluorescence microscopy. Quantification of autophagosome-like LC3B labeled dots was performed by measuring dot intensity with the Image J program and normalized as a percentage of the total cells present in a field as counted by the total number of nuclei (DAPI+). Then, 30–40 cells per image were analyzed; *n* = 10 images from 2 different cultures, per each condition. Data are expressed as mean ± SEM. Statistical analysis was done by Student’s *t*-test. Differences were accepted as significant if *p* < 0.05.

### 2.5. RT-PCR Analysis

#### 2.5.1. RNA Extraction

The RNeasy Mini Kit from Applied Biosystems (Thermo Fisher Scientific, Madrid, Spain) was used for total RNA isolation. Reverse transcription (RT) was carried out for one hour at 55 °C with oligodeoxythymidylate primer, using 5 μg of total RNA from each sample for complementary DNA synthesis. Real-time quantitative PCRs were performed in order to determine the levels of Beclin 1 (Becn1) and BNIP3, as well as housekeeping β-actin (β-Act) mRNAs by using the following specific primers synthesized at Sigma-Aldrich Co. (Madrid, Spain) (Table 2).

#### 2.5.2. Real-Time q-PCR

The SYBR Green PCR Master Mix (Applied Biosystems, Thermo Fisher Scientific, Madrid, Spain) and the 7900 HT Fast Real-Time PCR system (Applied Biosystems, Thermo Fisher Scientific, Madrid, Spain) were used to detect the real-time quantitative PCR products of the reverse-transcribed cDNA samples, according to the manufacturer’s instructions. q-PCR conditions were 95 °C (10 min), followed by 40 cycles of 15 s at 95 °C and annealing for 1 min at 60 °C. Three independent quantitative PCR assays (RT+) were performed for each gene and measured in triplicate. Three no-template controls (NTCs) were run for each quantitative PCR assay, and genomic DNA contamination of total RNA was controlled using three RT-minus controls (RT−) (samples without the reverse transcriptase).

Results are expressed as relative quantification (RQ), which was calculated from cycle thresholds (CTs) from each studied gene and housekeeping gene by means of the equation:

RQ = 2^−Δ(ΔCt)^, where Δ(ΔCt) = ΔCt(target gene) − ΔCt(calibrator gen) (1)

### 2.6. Cell Viability Test XTT

The XTT test (Cell Proliferation Kit II (XTT), Roche Diagnostics GMBH, Mannheim, Germany) is a colorimetric assay that allows nonradioactive spectrophotometric quantification of cell growth and viability without the use of radioactive isotopes. It is based on the ability of the cells to break the tetrazolium salts (yellow) by the action of mitochondrial dehydrogenases to form salts of formazan (orange), soluble in aqueous solutions, which are quantified spectrophotometrically. Therefore, an increase in the number of viable living cells reflects an increased activity of the mitochondrial dehydrogenase, which is directly correlated to the amount of orange formazan salt formed, increasing the absorbance of the sample.

Cells were seeded in 48-wells plates at a concentration of 250,000 cells in 500 μL/well of DMEM F12-Glutamax supplemented with 1% B27. In this medium, cells were incubated with the appropriate drugs for 24 h. Then, the medium was removed from the culture, and the cells were washed twice with fresh DMEM F12-Glutamax without B27-supplement. After this, 100 μL/well of yellow XTT solution (prepared by mixing 5 mL of the XXT labeling reagent and 0.1 mL of the electron coupling reagent, as described in the kit protocol, at a concentration 0.3 mg/mL) was added to the cells in a final volume of 200 μL of medium, and incubation was carried out for a maximal time of 4 h. During this time, absorbance measurements were performed at a time interval of 1 h. Spectrophotometric quantification was performed on a Biotek Power-Wave XS spectrophotometer microplate-reader (BioTek Instruments, Madrid, Spain) at a wavelength of 490 nm, using as reference a wavelength of 690 nm. Fitting of the concentration/response curves for estimation of EC_50_ values was made by weighted nonlinear regression of minimum squares, using logistic curves.

### 2.7. Caspase 3 Activity Test

Cells were seeded in 48-well plates in 500 μL of DMEM F12-Glutamax at a concentration of 250,000 cells/well. After a week of growth, control plates were changed with fresh culture medium, while experimental plates were changed with glucose-free medium and subjected to OGD for 3 h. Then, cells were put in the DMEM F12 Glutamax, glucose-containing medium and exposed to the treatments with drugs for 24 h. The protective effects of 1 μM phytoestrogens (1 μM) on apoptosis induced by OGDR were studied. In the control plates, the effects of culture medium alone (C24h) and DMSO (10%, 1%, 0.1%, 0.01%), which was used to dissolve the drugs, were studied. After 24 h of exposition to the treatments in a 95% air 5% CO_2_ atmosphere, the medium was removed, and the wells were washed with 300 μL of PBS. Then, 200 μL of caspase lysis buffer (10 mM Tris-HCl, 10 mM NaH_2_PO_4_/Na_2_HPO_4_, pH 7.5, 130 mM NaCl, 0.5% Triton X-100, 10 mM Na_4_P_2_O_7_, and 2 mM dithiothreitol (DTT) were added to the cells, and they were scraped. The suspension was put in an eppendorf tube and left in ice for 15 min. Finally, after centrifugation for 10 min at 13,000 rpm, the supernatant was stored at −80 °C.

To test the activity of caspase 3, the substrate N-acetyl-Asp-Glu-Val-AMC (7-amino-4-methylcoumarin) was used. It is stored lyophilized in PBS, and was prepared by adding 1 mL of water per mg. The buffer used for protease assay was 10 mM HEPES (pH 7.5, Sigma Aldrich, Madrid, Spain), 10% glycerol, and 2 mM DTT. The DTT was added just before using the buffer.

Caspase-3 activity was measured in the control and treated cortical neurons supernatants. Supernatants with at least 20 μg of protein (10–100 μL of lysates) were incubated at 37 °C for 2–4 h in 1 mL of caspase-3 assay buffer (20 mM HEPES, pH 7.5, 10% glycerol, 2 mM DTT) containing 20 μM Ac-DEVD-AMC [N-acetyl-Asp-Glu-Val-Asp-(7-amino-4-methylcoumarin)]. The fluorogenic AMC liberated from Ac-DEVD-AMC was monitored using a spectrofluorometer (Bio-Tek FL 600, BioTek Instruments, Madrid, Spain), with an excitation wavelength of 360 nm and an emission wavelength range of 460 nm. Under these conditions, the emission was linear between, at least, 0–6 h, depending on the caspase-3 activity. Enzymatic activity is expressed as arbitrary fluorescence units (AFU) after 1 h per mg protein (AFU/h/mg protein).

### 2.8. Statistics

Data were expressed as means ± SEM values of three or four independent experiments with different cell batches, each of which was performed in duplicate or triplicate. Statistical comparisons were performed by using one-way analysis of variance (ANOVA), followed by Holm−Sidak’s post-test when the analysis of variance was significant. Differences were accepted as significant if *p* < 0.05. Statistical analyses and fit curves for EC_50_ determinations were carried out using SigmaPlot 11.0 software (Systat Software, Inc., Erkrath, Germany). Statistical analysis for immunocytochemistry was done by Student’ *t*-test. Differences were accepted as significant if *p* < 0.05.

## 3. Results

### 3.1. Early Autophagy Activation Plays a Protective Role in Primary Cortical Neurons upon Experimental OGD and OGDR

**Figure 1:**
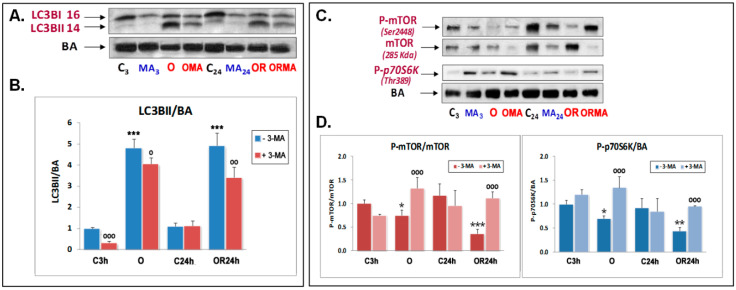
Effect of oxygen and glucose deprivation (OGD), oxygen and glucose deprivation and resupply (OGDR), and 5 mM 3-methyladenine (3-MA) treatment on LC3B lipidation (**A**,**B**) and mTOR and p70S6K phosphorylation (**C**,**D**) in cultured cortical neurons. (**A**,**B**) Western blot analysis of LC3BI and LC3BII was performed in extracts of cortical neurons subjected to 3 h OGD exposure (O) and subsequent 24 h OGDR (OR) compared to their respective controls. (**A**) Representative LC3B I and II and β-actin (BA) Western blots are shown. (**B**) Quantitative analysis of the LC3BII/BA ratio. Data are expressed as ratios over basal control and are mean ± SEM of three experiments, each one performed in duplicate. (**C**,**D**) Western blots of mTOR, ^2448^SerP-mTOR, and ^389^ThrP-p70S6K were performed in the aforementioned conditions. Upper panel (**C**) shows a representative Western blot of these proteins. Lower panels (**D**) shows the quantification of the indicated ratios and are mean ± SEM of three experiments, each one performed in duplicate. Statistics in panels (**B**) and (**D**) compare the effect of O and OR over their respective controls C3 and C24h, respectively (***) and the effect of 5 mM 3-MA in each condition, in the absence or presence of this compound (^ooo^), at *^/o^ *p* < 0.05, **^/oo^ *p* < 0.01, and ***^/ooo^ *p* < 0.001) (one-way ANOVA test).

Previous experiments of our research group suggested the involvement of different cell death pathways in OGD-induced neuronal death, including necrosis, apoptosis, and autophagy [29]. To determine if autophagy activation represents a pro-survival or a pro-death response during OGD damage, we tested the ability of 3-MA, a well-established inhibitor of autophagy initiation based on its inhibitory effect on class III PI3K activity, which is known to be essential for induction of autophagy [35], to affect cell death of primary neurons subjected to 3 h OGD and subsequent OGDR. First, Western blot analysis of LC3B, which is a well-documented indicator of autophagy activation [36], was carried out. LC3B data were normalized against β-Actin (BA) as a housekeeping gene, instead of microtubule-associated protein light chain 3B-I (LC3B-I), as this is currently considered as a more accurate autophagy indicator [37]. Results from Figure 1A,B show that OGD and OGDR induced an increase in LC3BI lipidation, which is reflected by the significant increase in microtubule-associated protein light chain 3B-II (LC3B-II/BA) ratio in both conditions. 3-MA had a small or nonsignificant effect on controls but, as expected, significantly reduced the increase in LC3B-I lipidation upon OGD and OGDR (Figure 1A). Therefore, these results demonstrated that 3-MA efficiently decreases OGD- and OGDR autophagy induction in primary cortical neurons.

In addition, we performed fluorescence image analysis of LC3B and found that LC3B-labeled dots increased upon OGDR compared to control cells (182.04 ± 12.93% and 100.71 ± 9.25%; mean ± SEM, for OGDR and C24h, respectively, *p* < 0.01 Student’s *t* test; Figure S1A,B). 3-MA treatment markedly diminished LC3B labeling versus the OGDR condition alone (76.73 ± 8.92%; *p* < 0.001 Student’s *t* test; Figure S1C).

It is well-known that autophagy is canonically inhibited when mTOR is activated by phosphorylation at ^2448^Ser. Consequently, when mTOR is inactive, autophagy can be engaged [6]. This autophagy-activating mechanism was also studied upon experimental OGD and OGDR conditions. We found that the phosphorylation of both ^2448^SerP-mTOR and ^389^ThrP-p70S6K, a well-known downstream substrate of mTOR that regulates protein synthesis [38], decreased upon OGD and/or OGDR, which are largely reverted by 3-MA (Figure 1C,D). Overall, these results show that autophagy activity becomes enhanced upon early OGD and is maintained during OGDR, both of which are abolished upon 3-MA treatment. 

**Figure 2:**
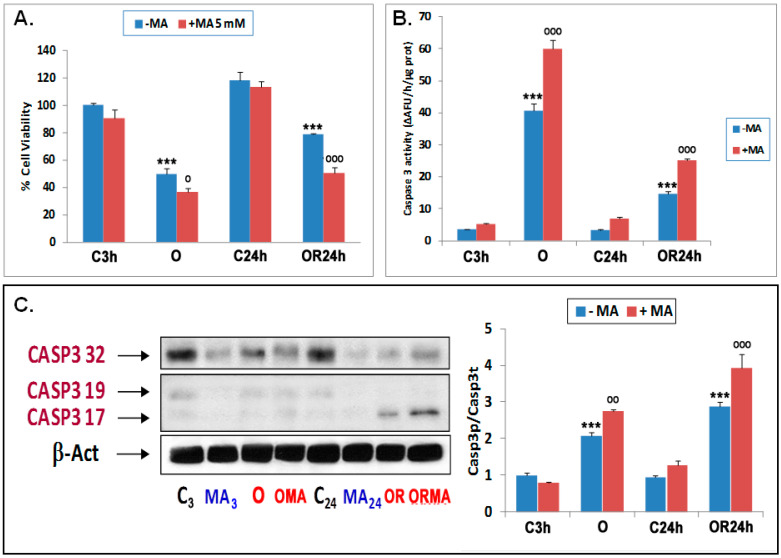
Effect of 3-methyladenine (3-MA) on cell viability and apoptosis of cortical neuron cultures under OGD and OGDR conditions. (**A**) Effect of 3-MA on cell viability measured by XTT assay. (**B**) Effect of 3-MA on caspase-3 activity. Both measurements were performed in extracts of cortical neurons subjected to 3 h OGD exposure (O) and 24 h OGDR (OR). Basal caspase-3 activity (C3h) was 3.58 ± 0.05 ΔAFU/h/µg protein. (**C**) Effect of 5 mM 3-MA on caspase-3 proteolysis in cortical neuron cultures under control conditions (C_3_ and C_24_) and OGD (O) and OGDR (OR) treatments. Western blots (CASP3 32 kDa; tCasp3 = total caspase 3) and cleaved (CASP3 19,17 kDa; pCasp3 = proteolyzed caspase 3) were performed in extracts of cortical neurons subjected to 3 h OGD exposure and 24 h OGDR, using two different anti CASP3 antibodies. Left: Representative CASP3 Western blots are shown. Right. Quantification of Western blot results showing the ratios between Casp3p = proteolyzed caspase and Casp3t = total caspase 3. Data are expressed as ratios over basal control and are mean ± SEM of three experiments, each of which was performed in triplicate (**A**,**B**) or duplicate (**C**). Statistics compare the effect of O and OR versus their respective controls (***) or the effect of 5 mM 3-MA under each condition versus respective conditions in the absence of this compound (^ooo^) at ^o^ *p* < 0.05, ^oo^ *p* < 0.01, ^ooo^ *p* < 0.001; one-way ANOVA test) are shown. C3h: control 3h; C24h: control 24h; OR24h: OGDR 24h; OMA: OGD plus 3-MA; ORMA: OGDR plus 3-MA; C_3_: control 3h; MA_3_: 3-MA 3h; MA_24_: 3-MA 24h.

To determine whether these autophagy changes represent a pro-survival or pro-death effect, we next investigated the effect of 3-MA on cultured neuron viability subjected to OGD and OGDR. First, we compared the metabolic activity of primary neurons subjected to control, OGD, or OGDR conditions using the XTT assay and found that OGD strongly diminished metabolic activity, which was partially mitigated in OGDR conditions (Figure 2A).

Moreover, while 3-MA treatment had no statistically significant effect in control conditions, it significantly decreased metabolic activity upon both OGD and OGDR, suggesting the initiation of autophagy plays a protective role. Second, we found that the activity of caspase 3, a key mediator of apoptosis, increased upon both OGD and OGDR, and was further exacerbated by 3MA (Figure 2B). To further confirm this result, we measured the enzymatically active proteolyzed forms of caspase 3 and found that 3-MA also exacerbated the production of activated caspase-3 induced by OGD and OGDR (Figure 2C). Altogether, these results show that the inhibition of autophagy initiation increases apoptosis under OGD conditions and, therefore, supports a pro-survival effect for early autophagy activation that protects OGD-subjected neurons from apoptotic cell death.

### 3.2. Phytoestrogens Afford Neuroprotection by Modulating Autophagy

**Figure 3:**
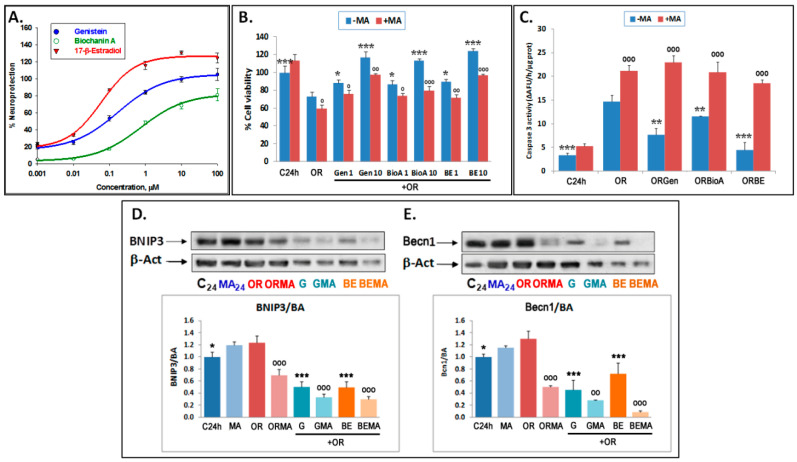
Neuroprotective effect of phytoestrogens on the decrease in cell viability, apoptosis, and autophagy (BNIP3 and Beclin 1 expression) induced by OGDR in cortical neuron cultures: Effect of 3-MA. (**A**) Dose–response curves of the neuroprotective effect afforded by 0.001–100 μM concentrations of phytoestrogens on the decrease in neuronal viability (metabolic activity) induced by OGDR (OR). The data are the means ± SEM of three determinations, each one performed in triplicate. Data analysis was performed with SigmaPlot 11 (see Section 2). (**B**) Effect of 5 mM 3-MA on the neuroprotective effect of Gen, BioA, and 17βE at 1–10 μM concentrations. Cell viability (metabolic activity) was measured by the XTT method. Data are expressed as percentages of cell viability of basal controls (100%) and are mean ± SEM of three experiments, each of which was performed in triplicate. In (**B**) the statistics compare the effect of the indicated compounds over OR alone (***) or the effect of 5 mM 3-MA treatments over their respective controls in the absence of this compound (^ooo^) at ^o^ *p* < 0.05, ^oo^ *p* < 0.01, ^ooo/^*** *p* < 0.001 (one-way ANOVA test). (**C**) Neuroprotective effect of phytoestrogens on the increase in apoptosis induced by OGDR. Effect of 5 mM 3-MA on the neuroprotective effects of Gen, BioA, and BE at 1 μM concentration. CASP 3 activity was measured as indicated in Material and Methods. Data are expressed as ΔAFU/h/μg protein and are mean ± SEM of three experiments, each of which was performed in triplicate. Statistics comparing the effect of indicated compounds over OR condition alone (***) or the effect of 5 mM 3-MA treatments over their respective controls in the absence of this compound (^ooo^) at ** *p* < 0.01, ***^/ooo^ *p* < 0.001 (one-way ANOVA test) are shown. (**D**,**E**) Effects of 1 μM genistein (Gen) or 17-β-estradiol (BE) on the expression of BNIP3 and Becn1 proteins. Upper panels show representative Western blots and lower panels represent the quantitative analysis of data expressed as ratios over basal control and are mean ± SEM of three experiments, each of which was performed in duplicate. Statistics comparing the effect of OR alone over its control and in the presence of phytoestrogens (***) and the effect of 5 mM 3-MA in each condition versus respective controls in the absence of this compound (ooo) (* *p* < 0.05, ^oo^ *p* < 0.01, ***/ooo *p* < 0.001; one-way ANOVA test) are shown. G: 1 μM genistein; GMA: 1 μM genistein plus 3-MA; BE: 1 μM 17-β-estradiol; BEMA: 1 μM 17-β-estradiol plus 3-MA.

To characterize the neuroprotective effect of the phytoestrogens in experimental OGDR, these phytoestrogens were added following OGD. Firstly, the effects of different doses of phytoestrogens on cell viability, on their own or in combination with 3-MA, were tested. The phytoestrogens used were Gen, and BioA, while BE was used as a reference compound as it is a pure agonist of estrogen receptors (ERs). Exposure to 0.001–100 µM of these compounds within 24 h of OGDR significantly reverted the OGDR-induced decrease in metabolic activity in a concentration-dependent manner (Figure 3A). The analysis of the % neuroprotection curves at the indicated concentrations revealed EC_50_ = 0.164 ± 0.016, 0.381 ± 0.017, and 0.062 ± 0.003 µM for Gen, BioA, and BE, respectively, indicating that the order of neuroprotective potency of these compounds is BE > Gen > BioA (*p* < 0.01, one-way ANOVA test). In addition, 3-MA treatment significantly reduced the neuroprotective effects of phytoestrogens and BE (Figure 3B), suggesting that phytoestrogens modulate autophagy to afford neuroprotection. In agreement with these findings, the phytoestrogens were able to significantly decrease caspase 3 activity induced by OGDR, the order of potency being BE > BioA > Gen. However, this antiapoptotic effect was dramatically abolished on exposure to 3-MA (Figure 3C). Altogether, these results indicate that the phytoestrogens, similar to BE, are neuroprotective upon OGDR conditions and that this effect is dependent on their modulation of autophagy initiation.

To determine whether the phytoestrogens modulate the expression levels of canonical autophagic proteins to afford neuroprotection, we measured the protein levels of the autophagy-initiating proteins Beclin1 and BNIP3 [39]. We decided to focus on Gen as this is a well-characterized phytoestrogen and because both Gen and BioA had a similar neuroprotective effect (Figure 3A–C).

Gen and BE treatment reduced the expression of Beclin-1 and BNIP3 at 24 h of OGDR, which was further enhanced by 3-MA treatment (Figure 3D,E). As explained in the discussion section, these inhibitory effects of phytoestrogens could be due to the fact that the mRNA expression of these autophagy-regulators genes was variable throughout time, such that it increased at 6 and 12 h after OGDR initiation in the case of Becn1 and BNIP3 mRNA expression, respectively, yet it decreased later on at 24 h of OGDR (Figure S2A,B). The differences in the times observed for the activating effects of phytoestrogens in the case of Becn1 and BNIP3 may be due to the fact that the mRNA levels of Becn1 drop more slowly than those of BNIP3 during OGDR, with the activating effect of the phytoestrogens being observed at 6 h of oxygen and glucose resupply, while in the case of BNIP3, the levels of mRNA expression decrease sharply after 6 h of OGDR and slightly recover at 12 and 24 h, and the activating effect of phytoestrogens is observed later at 12 h of OGDR. However, the inhibitory effect of phytoestrogens is seen in both cases at long OGDR times (24 h), when the mRNA expression levels of both genes are lower.

Collectively, these results indicate that the phytoestrogens positively modulate autophagy initiation early during OGDR, whereas at longer times, at least after 24 h of OGDR, phytoestrogens act as mild inhibitors of autophagy initiation. As further explained in the discussion section, these results suggest that autophagy may play a dual role during OGDR and that lowering autophagy at longer OGDR times is beneficial.

### 3.3. Phytoestrogens Activate mTOR and Act as MEK Inhibitors in Advanced Stages of OGDR

**Figure 4:**
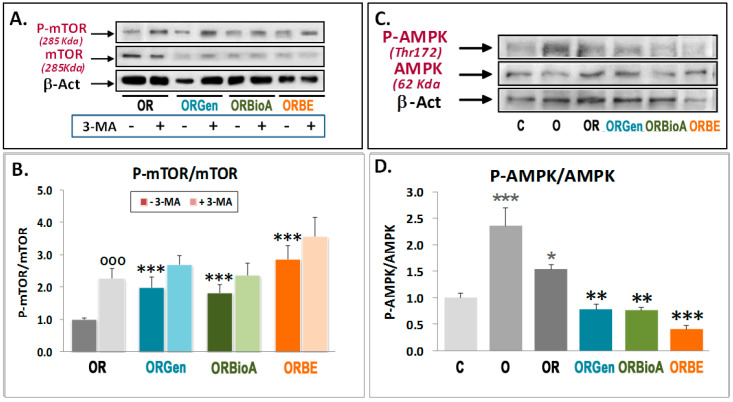
Effects of OGDR, phytoestrogens, and 3-MA treatment on mTOR and AMPK phosphorylation. Western blots of mTOR, ^2448^SerP-mTOR (**A**,**B**), and ^172^Thr-P-AMPK (**C**,**D**) were performed in extracts of cortical neurons exposed to 3 h OGD exposure (O) and 24 h OGDR (OR) in the absence or presence of 5 mM 3-MA (**A**,**B**) or under OR conditions in the absence or presence of 1 μM Genistein (Gen), BiocaninA (BioA), and 17-β-estradiol (BE)). Upper panels (**A**,**C**) show representative Western blots. Lower panels (**B**,**D**) represent the quantitative analysis of indicated ratios and are mean ± SEM of three experiments, each one performed in duplicate. Statistics comparing the effect of 5 mM 3-MA in each condition versus respective conditions in the absence of this compound (^ooo^) panel (**B**), the effect of O and OR over control C3 (***) panel (**D**), and the effect of phytoestrogens on OR condition without them (***) panels (**B**,**D**), at * *p* < 0.05, ** *p* < 0.01, and *** *p* < 0.001 (one-way ANOVA test) are shown.

We found that autophagy increases during OGDR conditions, which is concomitant with decreased mTOR activity (Figure 1C,D). Since phytoestrogens lower autophagy initiation after 24 h of OGDR (Figure 3D,E), we next tested whether this was linked to changes in mTOR activity. Treatment with Gen increased ^2448^Ser-mTOR phosphorylation (Figure 4A,B). To exclude that this was a Gen-specific effect, we also tested the effect of BioA and BE as a positive control on mTOR phosphorylation during OGDR, and found that both compounds also increased ^2448^Ser-mTOR phosphorylation (Figure 4A,B). In combination with 3-MA, there was no further increase in ^2448^Ser-mTOR phosphorylation, suggesting that both 3-MA and the phytoestrogens can activate mTOR with similar efficiency (Figure 4A,B). Altogether, these results indicate that phytoestrogens can prime mTOR to be more active in the late stages of OGDR, which is probably directly linked to their effect on autophagy-initiating proteins and their neuroprotective properties.

In order to know if the AMP-activated protein kinase (AMPK), a sensor of intracellular energy which inhibits mTOR phosphorylation, is involved in phytoestrogens-induced mTOR activation, the effect of OGDR and Gen, BioA, and BE was analyzed. As shown in Figure 4C,D, there was an increase in AMPK phosphorylation in OGD conditions, which was significantly reduced during OGDR, as expected. In these conditions, Gen, BioA, and BE were able to further reduce AMPK phosphorylation induced by OGDR, indicating that this pathway could be involved upstream in the inhibition of mTOR/S6K phosphorylation not only during OGDR but also as part of the neuroprotective effect of phytoestrogens.

**Figure 5:**
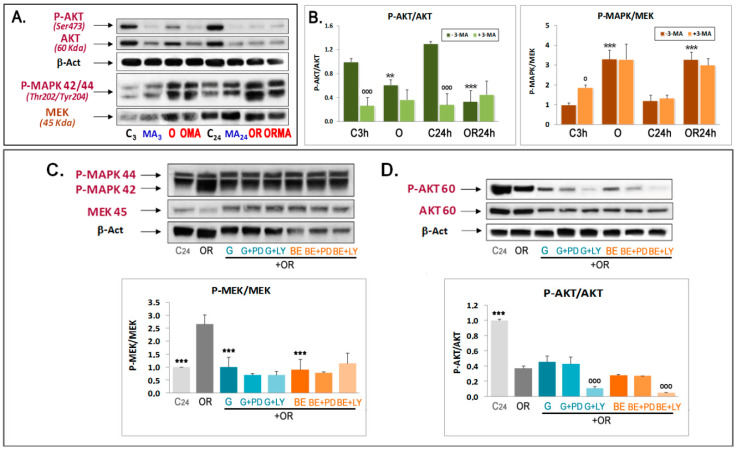
Effects of OGDR, phytoestrogens, and 3-MA treatment on AKT and MAPK phosphorylation. Western blots of ^473^SerP-AKT (**A**,**B**,**D**) and ^202^P-Thr- and ^204^P-Tyr-P-MAPK 42,44 (**A**–**C**) were performed in extracts of cortical neurons subjected to 3 h OGD exposure (O) and 24 h OGDR (OR), in the absence or presence of 5 mM 3-MA (**A**,**B**), or in the presence of 1 μM Gen or BE with or without 5 μM PD98059 or 5 μM LY294002 (**C**,**D**). Upper panels in (**C**,**D**) show representative Western blots. Lower panels represent the quantitative analysis of indicated ratios and are mean ± SEM of three experiments, each one performed in duplicate. Statistics in panel B compare the effect of OR over their respective controls (***) or the effect of 3-MA in each condition versus respective conditions in the absence of this compound (ooo). Statistics in panels (**C**,**D**) compare the effect of OR alone versus C24h and after addition of phytoestrogens (***) or the effect of PD or LY in each condition versus the same conditions in the absence of these compounds (^ooo^) at ^o^ *p* < 0.05, ** *p* < 0.01, *** *p* < 0.001 (one-way ANOVA test).

We also investigated the involvement of the AKT and MEK pathways as upstream mTOR regulatory pathways in the effect of phytoestrogens, for which we again focused on the effects of Gen and BE. To this end, ^473^SerP-AKT was used as the active form of this kinase [40] and p-MAPK (p44/42)(Thr^202^/Tyr^204^) as an indicator of MEK activity. We found that the pAKT/AKT ratio decreases under OGD and OGDR conditions compared to controls (Figure 5A,B). In addition, 3-MA treatment inhibited AKT phosphorylation in controls, yet it remained unchanged upon OGD and OGDR (Figure 5A,B). Regarding the MEK/ERK1/2 pathway, both OGD and OGDR increased MAPK 42/44 phosphorylation, and 3-MA did not have any effect under OGD conditions (Figure 5A,B). Altogether, these results indicate that while the ERK1/2 pathway becomes activated under OGD conditions, the PI3K/AKT pathway is dampened down, which collectively underlies the subsequent downstream decrease in mTOR/S6K activation and autophagy activation. 

Although Gen and BE treatments increased mTOR phosphorylation, they did not have any significant effect on the inhibition of AKT phosphorylation induced by OGDR (Figure 5D). This was not a detection problem as we detected decreased pAKT after cotreating with the PI3K inhibitor LY294002 (LY) but not with the MEK inhibitor PD98059 (PD) (Figure 5D). Interestingly, Gen and BE significantly reversed the increase in MAPK phosphorylation induced by OGDR, which was not further reduced by PD treatment, thus indicating a very strong effect of phytoestrogens on MAPK phosphorylation (Figure 5C). Therefore, phytoestrogens behave as classical MAPK inhibitors rather than classical activators of AKT phosphorylation. 

## 4. Discussion

Autophagy is an important biological mechanism implicated in the regulation of numerous fundamental cellular processes by enhancing the turnover of nonfunctional proteins and organelles [41]. Neuronal cells, like other eukaryotic cells, are dependent on autophagy for neuroprotection in response to stress, but autophagy can also induce cell death in cerebral ischemia [42]. Recent studies have demonstrated that autophagy may induce neuroprotection following acute brain injury, including ischemic stroke [9,43]. However, in some special circumstances, activation of autophagy can induce cell death, playing a deleterious role in the etiology and progression of ischemic stroke [9,43,44]. Currently, there are no therapeutic options against stroke that demonstrate efficient neuroprotective abilities. For this reason, more indepth mechanism research is still needed about autophagy and its potential use as a new therapeutic target for stroke [44,45]. 

In the present work, we have examined the contribution of autophagy in the context of OGD and OGDR by first outlining its role in OGD-induced neuronal death. In addition, we have characterized the cellular signaling pathways modulated by two naturally occurring phytoestrogens in regards to their neuroprotective effects in this in vitro model and autophagy to assess their possible therapeutic application in stroke. 

Firstly, we addressed whether autophagy activation, a well-known event in experimental ischemia [29], plays a pro-survival or a pro-death role in OGD–OGDR-induced neuronal damage. We found that an OGD-induced decrease in cell viability and an increase in caspase-3 activity, a well-known marker of apoptosis activation, were further potentiated on exposure to the autophagy inhibitor 3-MA [35]. These results, along with the increase observed in lipidation of LC3I upon OGD and OGDR, showed that autophagy activation is part of the endogenous pro-survival response to these neuronal insults to protect neurons from enhanced apoptotic cell death upon OGD and OGDR. 

Once we established the pro-survival role of autophagy in OGD and OGDR, we explored whether the neuroprotective effects of phytoestrogens previously observed by us [29] and other laboratories in in vitro [25] and in vivo models of cerebral ischemia [21,26,27,46,47] were related to autophagy. First, we confirmed the neuroprotective properties of phytoestrogens by showing that phytoestrogen treatment strongly ameliorated metabolic activity and reduced caspase-3 activation (i.e., apoptosis activation) after OGDR. However, this neuroprotection was blocked upon 3-MA cotreatment. Given that 3-MA is an autophagy inhibitor, our results suggest that autophagy activation is required for phytoestrogens to exert a neuroprotective effect and protect against OGDR-induced apoptosis. 

To further confirm the activation of autophagy in OGD and OGDR conditions, the expression levels of the canonical autophagy-initiator proteins Beclin 1 and BNIP3, which are transcriptionally induced by the hypoxia-inducible factor (HIF1α) activation during ischemia [48], were analyzed in our experimental setting. Both BNIP3 and Beclin-1 were initially identified as BCL2 or BCL-XL-interacting proteins. Beclin-1 is part of a class III PI3-K containing complex that regulates autophagy induction, and BCL2 inhibits autophagy by binding to Beclin-1. BNIP3 competes with Beclin-1 for binding to BCL-XL, and its increased expression in hypoxia could disrupt pre-existing BCL-XL-Beclin-1 complexes, thereby releasing Beclin-1 and activating autophagy [49]. We found a significant increase in Beclin-1 expression at the protein level upon OGDR, along with a mild but significant increase in BNIP3 levels. Of note, the mild changes in BNIP3 protein levels upon OGDR are in agreement with our data showing that the increased expression of BNIP3 at the mRNA level occurs very transiently in OGD and decreases rapidly during OGDR, unlike the mRNA expression of Beclin 1 which decreases more slowly (similarly to those of HIF1α; data not shown), as previously shown [29]. Surprisingly, treatment with Gen and BE during the period of oxygen and glucose resupply reduced Beclin-1 and BNIP3 levels, this effect being more potent in association with 3-MA. These results indicate that while autophagy activation is required for the neuroprotective effect of phytoestrogens, these lower autophagy at later stages, at least as late as 24 h of treatment during OGDR. In agreement with these results, we found that phytoestrogens increase Becn1 and BNIP3 mRNA levels at short periods of OGDR, while at longer times, phytoestrogens lower the transcriptional levels of these two autophagic proteins. Therefore, we propose that phytoestrogens exert a dual modulation on autophagy upon OGDR, depending on the time point. Alternatively, phytoestrogens could reduce mitochondrial protein levels such as Becn1 and BNIP3 as a simple off-target effect or act through mitophagy [49,50]. Future studies should address whether chronically activating autophagy is detrimental in ischemic conditions and also if it prevents the long-term neuroprotective effects of phytoestrogens. 

Recent studies on the expression of these genes, at the mRNA level, in models of rats with ischemia or Alzheimer’s disease with ischemia [51,52,53] have also demonstrated great variability in the expression of these autophagic and mitophagic genes in CA1 [51] and CA3 [52] regions of the hippocampus, as well as in temporal lobe of the cerebral cortex [53]. In the latter, marked differences were reported between the expression of Becn1 and BNIP 3 after a temporal course of 30 days after ischemia—that of Becn1 being high at the beginning, with a tendency to decrease during the 30 days of the reperfusion period, and that of BNIP3 having a tendency to ascend in the mid-term and then to decrease. Although this model is not comparable to our in vitro model, changes in the expression of these genes at the mRNA level are likely to influence the levels of these proteins involved in autophagy and mitophagy, thus playing a relevant role in the response to brain ischemia in the cortex and hippocampus. In this regard, measuring the expression of these genes at protein level at different times of OGDR and/or the autophagic flux using a lysosomal inhibitor, such as bafilomycin or chloroquine, would provide interesting information regarding the time-dependent effect of OGD, OGDR, and phytoestrogens on autophagy.

Furthermore, we explored the potential involvement of mTOR/p70S6K as an important part of the autophagy inhibition pathway [54] and its possible regulators (PI3K/AKT, MAPK, or AMPK) in OGD and OGDR, as well as in the neuroprotective effects of phytoestrogens. In keeping with previous results shown above, the protein levels of the active form of the autophagy blocker mTOR (^2448^SerP-mTOR) dropped upon OGD, and so did those of its well-known substrate ^389^ThrP-p70S6K. Of note, these effects were reverted by 3-MA. Overall, these results indicate that the mTOR/S6K pathway participates in both the induction of OGD-triggered autophagy and in the 3-MA-induced inhibition of OGD-triggered autophagy.

In the same way that the phytoestrogens inhibited the expression of proteins involved in the initiation of autophagy, Beclin 1 and BNIP3, phytoestrogens were found to increase mTOR-activating phosphorylation at 24 h of oxygen and glucose resupply treatment. Collectively, these results suggest that phytoestrogens could play a dual role in the regulation of autophagy by acting as “autophagy enhancers” when autophagy is a neuroprotective and pro-survival mechanism, and as “autophagy inhibitors” when autophagy is a pro-death mechanism. The last mechanism could be mediated in an mTOR-dependent manner. 

To determine whether phytoestrogens act upstream of mTOR to lead to enhanced mTOR activation at 24 h OGDR, we investigated their effect on the activated state of three well-documented pathways that modulate mTOR activity, namely, AMPK, AKT, and MEK. First, we investigated the AMPK pathway, which inhibits mTOR [55]. We observed an increase in AMPK phosphorylation under the OGD condition, which was significantly reverted during OGDR. Furthermore, phytoestrogens and BE reduced further OGDR-induced AMPK phosphorylation, indicating that this pathway is targeted by phytoestrogens upstream to trigger mTOR activation after 24 h of OGDR. Second, we found a remarkable decrease in AKT phosphorylation under OGDR conditions, which was unaffected by 3-MA treatment. In contrast, 3-MA strongly inhibited AKT phosphorylation under control conditions. While this effect could be related to an increase in neuronal death following autophagy inhibition, we did not find evidence of increased cell death upon 3-MA treatment in control conditions. Therefore, it is conceivable that other neuroprotective signaling pathways different to PI3K/AKT could be at play to protect control cells from death when autophagy is inhibited in basal conditions. Regarding the MAPK pathway, OGDR increased MAPK phosphorylation, although in this case, 3-MA did not have any effect. These results suggest that there is an opposite regulation by the AKT and MEK/ERK signaling pathways in OGD–OGDR, and that only the PI3K/AKT pathway is involved in OGDR-induced protective autophagy. However, these results could also be in agreement with a recent noncanonical MEK/ERK pathway downstream of AMPK and upstream of TSC and mTOR [56]. This MEK/ERK pathway regulates autophagy via regulating the Beclin 1 level through the AMPK-MEK/ERK-TSC-mTOR pathway.

Regarding the effect of phytoestrogens, Gen and BE did not have significant effects on the OGDR-induced inhibition of AKT phosphorylation, yet they largely reversed the OGDR-induced increase in MAPK phosphorylation. However, since the reversion of MAPK phosphorylation was not abolished by PD98059, these data are not completely clear and need to be further clarified, although these data seem to suggest that MAPK inhibition, rather than AKT activation pathways, could be involved in the neuroprotective effects of phytoestrogens in OGDR.

Finally, both the Ras-ERK and PI3K-mTORC1 pathways represent key mechanisms for cells to regulate cell survival and proliferation and, in addition to their independent signaling programs that provide compensatory mechanisms, the pathways crosstalk extensively and regulate each other both positively and negatively [57]. Pathway convergence and AGC kinase promiscuity have been shown, and ERK and the AGC kinases, like AKT, often regulate the same substrates, yielding the same phenotypic effects [45]. However, because no effects of MAPK inhibitors nor AKT inhibitors have been observed in the effect of phytoestrogens on these pathways, our results seem to support that phytoestrogens activate mTOR by reducing MAPK activation through mechanisms involving the inhibition of Beclin-1 expression and AMPK phosphorylation rather than to a negative interaction between ERK and AGC kinases, like AKT.

Recently many authors have been interested in studying the possible crosstalk between different mechanisms of death in neurons, the current general consensus being that autophagy is initially activated as a neuroprotective mechanism against necrotic and apoptotic neuronal death in situations of brain damage such as ischemia, but that excessive accumulation of autophagosomes may become a mechanism of death in later stages when the autophagic stress increased [58]. Thus, autophagy would play a double role in the regulation of neuronal death. Our results support this hypothesis that indicates an activation of autophagy when apoptosis and necrosis are severely activated, as is the case with ischemia, and that when damage decreases with the time of reperfusion, autophagy may have a role in promoting cell death. More recently, autophagy has also been seen to crosstalk with other nonapoptotic or regulated necrotic death pathways such as necroptosis and pyroptosis [59], which have also been reported in brain ischemia [60]. Thus, this would be an interesting topic to consider in future studies. This knowledge will undoubtedly provide new therapeutic targets and new mechanisms to understand the brain damage physiopathology. Signaling mediated by trophic factors, hormones, and antioxidant and anti-inflammatory molecules, such as phytoestrogens, could be good examples of modulators of these processes as they could regulate different mechanisms that mediate neuronal demise, including necrosis, apoptosis, necroptosis, pyroptosis, and autophagy.

## 5. Conclusions

Overall, our findings provide renewed insight on the mechanism of action of autophagy in an in vitro model of OGD and OGDR injury together with the molecular mechanisms underlying the neuroprotective effects of phytoestrogens and estrogens, shedding light on the previously disputed therapeutic potential of autophagy modulators and supporting the indepth study of mild autophagy activators in in vivo models of ischemia [61].

## Figures and Tables

**Table 1:** Antibodies (Ab) used for western blot and immunocytochemistry assays.

Assay Type	Primary Ab	Concentration	Producer	MW (kDa)	Secondary Ab	Concentration	Producer
**(A) Western Blot**	Rabbit-anti LC3B	1/1000	Cell Signalling	16, 14	HRP-conjugated goat anti-rabbit	1/1000	Sigma-Aldrich
	Rabbit-anti caspase 3 Rabbit-anti-Cleaved-casp. 3	1/1000	Cell Signalling	32, 19, 17	HRP-conjugated goat anti-rabbit	1/1000	Sigma-Aldrich
	Rabbit-anti mTOR Rabbit-anti **^Ser2448^P**-mTOR	1/1000	Cell Signalling	285	HRP-conjugated goat anti-rabbit	1/1000	Sigma-Aldrich
	Rabbit-anti AMPKα Rabbit-anti **^Thr172^P**-AMPK	1/1000	Cell Signalling	62	HRP-conjugated goat anti-rabbit	1/1000	Sigma-Aldrich
	Rabbit-anti p70S6K Rabbit-anti **^Thr389^P**-p70S6K	1/1000	Cell Signalling	70, 85	HRP-conjugated goat anti-rabbit	1/1000	Sigma-Aldrich
	Rabbit-anti AKT Rabbit-anti **^Ser473^P**-AKT	1/1000	Cell Signalling	60	HRP-conjugated goat anti-rabbit	1/1000	Sigma-Aldrich
	Rabbit-anti **^Thr202/Tyr204^P**-MAPK42,44	1/1000	Cell Signalling	42, 44	HRP-conjugated goat anti-rabbit	1/1000	Sigma-Aldrich
	Rabbit-anti Beclin-1	1/1000	Cell Signalling	60	HRP-conjugated goat anti-rabbit	1/1000	Sigma-Aldrich
	Rabbit-anti BNIP3	1/1000	Cell Signalling	30	HRP-conjugated goat anti-rabbit	1/1000	Sigma-Aldrich
	Mouse-anti β-Actin	1/6000	Sigma-Aldrich	42	HRP-conjugated goat anti-mouse	1/4000	Sigma-Aldrich
**(B) Fluorescence Immunocytochemistry**	Rabbit-anti LC3B	1/300	Cell Signalling	16, 14	Donkey anti-rabbit Alexa Fluor 488	1/300	Sigma-Aldrich
	DAPI	1/1000	Sigma-Aldrich	277.3	-	-	-

MW: molecular weight; LC3B: microtubule-associated protein light chain 3B; DAPI: 4′,6-diamidino-2-phenylindole.

**Table 2:** Primers used for RTqPCR.

Primer	Sequence
*βAct* forward	5′-GCCAACCGTGAAAAGATGA-3′
*βAct* reverse	5′-TACGACCAGAGGCATACAGG-3′
*Becn1* forward	5′-GGCTCCTATTCCATCAAAACC-3′
*Becn1* reverse	5′-GGACACCCAAGCAAGACC-3′
*BNIP3* forward	5′-GCTGAAATAGACACCCACAGC-3′
*BNIP3* reverse	5′-GACTTGACCAATCCCATATCC-3′

βAct: β-Actin; Becn1: Beclin 1; BNIP3: Bcl2 and adenovirus E1B 19 kDa-interacting protein 3.

## Supplementary Materials

The following are available online at https://www.mdpi.com/2076-3921/9/6/545/s1. Figure S1: Effect of 3-MA on LC3B lipidation in cultured cortical neurons under OGD and OGDR conditions. Figure S2: Effects of phytoestrogens on Beclin 1 (Becn1) (A) and BNIP3 (B) mRNA expression in cultured cortical neurons under OGD and OGDR conditions.

## Abbreviations

Gen	Genistein
Becn1	Beclin 1
BioA	Biochanin A
Daid	Daidzein
BE	17β-estradiol
ER	Estrogen receptors
3-MA	3-Methyladenine
OGD	Oxygen and Glucose Deprivation
OGDR	Oxygen and Glucose Deprivation and Resupply
LY294002	AKT inhibitor
PD98059	MEK inhibitor

## References

[1] Castillo J., Alvarez-Sabin J., Dávalos A., Díez-Tejedor E., Lizasoain I., Martínez-Vila E., Vivancos J., Zarranz J.J. (2003). Consensus review. Pharmacological neuroprotection in cerebral ischemia: Is it still a therapeutic option?. Neurologia.

[2] Pluta R., Salínska E., Puka M., Stafiej A., Lazarewicz J.W. (1988). Early changes in extracellular amino acids and calcium concentrations in rabbit hippocampus following complete 15-min cerebral ischemia. Resuscitation.

[3] Hossmann K.A. (2006). Pathophysiology and therapy of experimental stroke. Cell. Mol. Neurobiol..

[4] Rami A., Kögel D. (2008). Apoptosis meets Autophagy-Like cell death in the ischemic penumbra: Two sides of the same coin?. Autophagy.

[5] Fernández-Gómez F.J., Hernández F., Argandoña L., Galindo M.F., Segura T., Jordán J. (2008). Farmacología de la neuroprotección en el ictus isquémico agudo. Rev. Neurol..

[6] Murrow L., Debnath J. (2013). Autophagy as a Stress-Response and Quality-Control mechanism: Implications for cell injury and human disease. Annu. Rev. Pathol..

[7] Nixon R.A. (2013). The role of autophagy in neurodegenerative disease. Nat. Med..

[8] Adhami F., Liao G., Morozov Y.M., Schloemer A., Schmithorst V.J., Lorenz J.N., Dunn R.S., Vorhees C.V., Wills-Karp M., Degen J.L. (2006). Cerebral Ischemia-Hypoxia induces intravascular coagulation and autophagy. Am. J. Pathol..

[9] Gabryel B., Kost A., Kasprowska D. (2012). Neuronal autophagy in cerebral ischemia–a potential target for neuroprotective strategies?. Pharmacol. Rep..

[10] Zhang J., Liu J., Li D., Zhang C., Liu M. (2019). Calcium antagonists for acute ischemic stroke. Cochrane Database Syst. Rev..

[11] Sarkar S., Chakraborty D., Bhowmik A., Ghosh M.K. (2019). Cerebral ischemic stroke: Cellular fate and therapeutic opportunities. Front. Biosci. (Landmark. Ed.).

[12] Ginsberg M.D. (2008). Neuroprotection for ischemic stroke: Past, present and future. Neuropharmacology.

[13] Chamorro Á., Dirnagl U., Urra X., Planas A.M. (2016). Neuroprotection in acute stroke: Targeting excitotoxicity, oxidative and nitrosative stress, and inflammation. Lancet Neurol..

[14] Radak D., Katsiki N., Resanovic I., Jovanovic A., Sudar-Milovanovic E., Zafirovic S., Mousad S.A., Isenovic E.R. (2017). Apoptosis and Acute Brain Ischemia in Ischemic Stroke. Curr. Vasc. Pharmacol..

[15] Moretti A., Ferrari F., Villa R.F. (2015). Neuroprotection for ischaemic stroke: Current status and challenges. Pharmacol. Ther..

[16] Gibson C.L., Gray L.J., Murphy S.P., Bath P.M. (2006). Estrogens and experimental ischemic stroke: A systematic review. J. Cereb. Blood Flow Metab..

[17] Etgen A.M., Jover-Mengual T., Zukin R.S. (2011). Neuroprotective Actions of Estradiol and Novel Estrogen Analogs in Ischemia: Translational Implications. Front. Neuroendocr..

[18] Bramlett H.M., Dietrich W.D. (2001). Neuropathological protection after traumatic brain injury in intact female rats versus males or ovariectomized females. J. Neurotrauma.

[19] Schreihofer D.A., Oppong-Gyebi A. (2019). Genistein: Mechanisms of action for a pleiotropic neuroprotective agent in stroke. Nutr. Neurosci..

[20] Shambayati M., Patel M., Ma Y., Cunningham R.L., Schreihofer D.A. (2014). Central inflammatory response to experimental stroke is inhibited by a neuroprotective dose of dietary soy. Brain Res..

[21] Castelló-Ruiz M., Torregrosa G., Burguete M.C., Salom J.B., Gil J.V., Miranda F.J., Jover-Mengual T., Marrachelli V.G., Alborch E. (2011). Soy-Derived phytoestrogens as preventive and acute neuroprotectors in experimental ischemic stroke: Influence of rat strain. Phytomedicine.

[22] Cortina B., Torregrosa G., Castelló-Ruiz M., Burguete M.C., Moscardó A., Latorre A., Salom J.B., Vallés J., Santos M.T., Alborch E. (2013). Improvement of the circulatory function partially accounts for the neuroprotective action of the phytoestrogen genistein in experimental ischemic stroke. Eur. J. Pharmacol..

[23] Schreihofer D.A., Do K.D., Schreihofer A.M. (2005). High-Soy diet decreases infarct size after permanent middle cerebral artery occlusion in female rats. Am. J. Physiol. Regul. Integr. Comp. Physiol..

[24] Schreihofer D.A. (2009). Phytoestrogens as neuroprotectants. Drugs Today (Barc.).

[25] Schreihofer D.A., Redmond L. (2009). Soy phytoestrogens are neuroprotective against Stroke-Like injury in vitro. Neuroscience.

[26] Ma T.C., Campana A., Lange P.S., Lee H.H., Banerjee K., Bryson J.B., Mahishi L., Alam S., Giger R.J., Barnes S. (2010). A Large-Scale chemical screen for regulators of the arginase 1 promoter identifies the soy isoflavone Daidzein as a clinically approved small molecule that can promote neuronal protection or regeneration via a cAMP-Independent pathway. J. Neurosci..

[27] Ma Y., Sullivan J.C., Schreihofer D.A. (2010). Dietary Genistein and equol (4,7 isoflavandiol) reduce oxidative stress and protect rats against focal cerebral ischemia. Am. J. Physiol. Regul. Integr. Comp. Physiol..

[28] Kim J., Fann D.Y., Seet R.C., Jo D.G., Mattson M.P., Arumugam T.V. (2016). Phytochemicals in ischemic stroke. Neuromol. Med..

[29] Arce C., Arteaga J.L., Sánchez-Mendoza E., Oset-Gasque M.J., Cañadas S., González M.P. (2010). Added after Anoxia-Reoxigenation stress, genistein rescues from death the rat embryo cortical neurons. Neurosci. Med..

[30] Hurtado O., Ballesteros I., Cuartero M.I., Moraga A., Pradillo J.M., Ramírez-Franco J., Bartolomé-Martín D., Pascual D., Torres M., Sánchez-Prieto J. (2012). Daidzein has neuroprotective effects through ligand-binding-independent PPAR γ activation. Neurochem. Int..

[31] Aras A.B., Guven M., Akman T., Alacam H., Kalkan Y., Silan C., Cosar M. (2015). Genistein exerts neuroprotective effect on focal cerebral ischemia injury in rats. Inflammation.

[32] Guo M., Lu H., Qin J., Qu S., Wang W., Guo Y., Liao W., Song M., Chen J., Wang Y. (2019). Biochanin a provides neuroprotection against cerebral Ischemia-Reperfusion injury by Nrf2-Mediated inhibition of oxidative stress and inflammation signaling pathway in rats. Med. Sci. Monit..

[33] Figueroa S., Oset-Gasque M.J., Arce C., Martínez-Honduvilla C., González M.P. (2006). Michocondrial involvement in nitric Oxide-Induced cellular death in cortical neurons in culture. J. Neurosc. Res..

[34] Ryou M.G., Mallet R.T. (2018). An in vitro Oxygen-Glucose deprivation model for studying ischemia-reperfusion injury of neuronal cells. Methods Mol. Biol..

[35] Wu Y.T., Tan H.L., Shui G., Bauvy C., Huang Q., Wenk M.R., Ong C.N., Codogno P., Shen H.M. (2010). Dual role of 3-methyladenine in modulation of autophagy via different temporal patterns of inhibition on class I and III phosphoinositide 3-kinase. J. Biol. Chem..

[36] Tanida I., Ueno T., Kominami E. (2004). LC3 conjugation system in mammalian autophagy. Int. J. Biochem. Cell Biol..

[37] Mizushima N., Yoshimori T. (2007). How to interpret LC3 immunoblotting. Autophagy.

[38] Tavares M.R., Pavan I.C., Amaral C.L., Meneguello L., Luchessi A.D., Simabuco F.M. (2015). The S6K protein family in health and disease. Life Sci..

[39] Lu N., Li X., Tan R., An J., Cai Z., Hu X., Wang F., Wang H., Lu C., Lu H. (2018). HIF-1α/Beclin1-Mediated autophagy is involved in neuroprotection induced by hypoxic preconditioning. Mol. Neurosci..

[40] Sale E.M., Sale G.J. (2008). Protein kinase B: Signalling roles and therapeutic targeting. Cell. Mol. Life Sci..

[41] Mizushima N., Komatsu M. (2011). Autophagy: Renovation of cells and tissues. Cell.

[42] Xu F., Gu J.H., Qin Z.H. (2012). Neuronal autophagy in cerebral ischemia. Neurosci. Bull..

[43] Mo Y., Sun Y.Y., Liu K.Y. (2020). Autophagy and inflammation in ischemic stroke. Neural Regen. Res..

[44] Hou K., Xu D., Li F., Chen S., Li Y. (2019). The progress of neuronal autophagy in cerebral ischemia stroke: Mechanisms, roles and research methods. J. Neurol. Sci..

[45] Nabavi S.F., Sureda A., Sanches-Silva A., Pandima Devi K., Ahmed T., Shahid M., Sobarzo-Sánchez E., Dacrema M., Daglia M., Braidy N. (2019). Novel therapeutic strategies for stroke: The role of autophagy. Crit. Rev. Clin. Lab. Sci..

[46] Altavilla D., Crisafulli A., Marini H., Espósito M., D’Anna R., Corrado F., Bitto A., Squadrito F. (2004). Cardiovascular effects of the phytoestrogen genistein. Med. Chem. Cardiovasc. Hematol. Agents.

[47] Burguete M.C., Torregrosa G., Pérez-Asensio F.J., Castelló-Ruiz M., Salom J.B., Gil J.V., Alborch E. (2006). Dietary phytoestrogens improve stroke outcome after transient focal cerebral ischemia in rats. Eur. J. Neurosci..

[48] Bellot G., Garcia-Medina R., Gounon P., Chiche J., Roux D., Pouysségur J., Mazure N.M. (2009). Hypoxia-induced autophagy is mediated through hypoxia-inducible factor induction of BNIP3 and BNIP3L via their BH3 domains. Mol. Cell. Biol..

[49] Zhang J., Ney P.A. (2009). Role of BNIP3 and NIX in cell death, autophagy, and mitophagy. Cell Death Differ..

[50] Shi R.Y., Zhu S.H., Li V., Gibson S.B., Xu X.S., Kong J.M. (2014). BNIP3 interacting with LC3 triggers excessive mitophagy in delayed neuronal death in stroke. CNS Neurosci. Ther..

[51] Ułamek-Kozioł M., Kocki J., Bogucka-Kocka A., Januszewski S., Bogucki J., Czuczwar S.J., Pluta R. (2017). Autophagy, mitophagy and apoptotic gene changes in the hippocampal CA1 area in a rat ischemic model of Alzheimer’s disease. Pharmacol. Rep..

[52] Ułamek-Kozioł M., Czuczwar S.J., Kocki J., Januszewski S., Bogucki J., Bogucka-Kocka A., Pluta R. (2019). Dysregulation of Autophagy, Mitophagy, and apoptosis genes in the CA3 region of the hippocampus in the ischemic model of Alzheimer’s disease in the rat. J. Alzheimer’s Dis..

[53] Ułamek-Kozioł M., Kocki J., Bogucka-Kocka A., Petniak A., Gil-Kulik P., Januszewski S., Bogucki J., Jabłoński M., Furmaga-Jabłońska W., Brzozowska J. (2016). Dysregulation of autophagy, Mitophagy, and apoptotic genes in the medial temporal lobe cortex in an ischemic model of Alzheimer’s disease. J. Alzheimer’s Dis..

[54] Ma X.M., Blenis J. (2009). Molecular mechanisms of mTOR-mediated translational control. Nat. Rev. Mol. Cell. Biol..

[55] Tamargo-Gómez I., Mariño G. (2018). AMPK: Regulation of metabolic dynamics in the context of autophagy. Int. J. Mol. Sci..

[56] Wang J., Whiteman M.W., Lian H., Wang G., Singh A., Huang D., Denmark T.A. (2009). Non-canonical MEK/ERK signaling pathway regulates autophagy via regulating Beclin 1. J. Biol. Chem..

[57] Mendoza M.C., Er E.E., Blenis J. (2011). The Ras-ERK and PI3K-mTOR pathways: Cross-talk and compensation. Trends Biochem. Sci..

[58] Adhami F., Schloemer A., Kuan C.Y. (2007). The roles of autophagy in cerebral ischemia. Autophagy.

[59] Nikoletopoulou V., Markaki M., Palikaras K., Tavernarakis N. (2013). Crosstalk between apoptosis, necrosis and autophagy. Biochim. Biophys. Acta.

[60] Tovar-y-Romo L.B., Penagos-Puig A., Ramírez-Jarquín J.O. (2016). Endogenous recovery after brain damage: Molecular mechanisms that balance neuronal life/death fate. J. Neurochem..

[61] Acaz-Fonseca E., Castelló-Ruiz M., Burguete M.C., Aliena-Valero A., Salom J.B., Torregrosa G., García-Segura L.M. (2020). Insight into the molecular sex dimorphism of ischaemic stroke in rat cerebral cortex: Focus on neuroglobin, sex steroids and autophagy. Eur. J. Neurosci..

