# Supplementary material for: Neuroprotection by Phytoestrogens in the Model of Deprivation and Resupply of Oxygen and Glucose In Vitro: The Contribution of Autophagy and Related Signaling Mechanisms
Source: Antioxidants (Basel). 2020 Jun 22;9(6):545. doi: 10.3390/antiox9060545 (PMC7346137; doi:10.3390/antiox9060545)
Supplement: Supplementary file 1 [file antioxidants-09-00545-s001.pdf]

Supplementary Materials

Article

Neuroprotection by Phytoestrogens in the Model of Deprivation and Resupply of Oxygen and Glucose In Vitro: The Contribution of Autophagy and Related Signaling Mechanisms

Giuseppe Abbruzzese [1], Javier Morón-Oset [1,†], Sabela Díaz-Castroverde [1], Nuria García-Font [1], Cesáreo Roncero [1,2], Francisco López-Muñoz [3,4]; José Luis Marco Contelles [5] and María Jesús Oset-Gasque [1,2,6,*]

[1] Department of Biochemistry and Molecular Biology, Faculty of Pharmacy, Complutense University of Madrid, E-28040 Madrid, Spain; giu.abbru@gmail.com (G.A.); javimoroset@gmail.com (J.M.-O.); sabelacastroverde@gmail.com (S.D.-C.); nuriagarciafont@ucm.es (N.G.-F.); ceronce@ucm.es (C.R.)

[2] Health Research Institute of the Hospital Clínico San Carlos (IdISSC), 28040 Madrid, Spain.

[3] Faculty of Health, Camilo José Cela University of Madrid (UCJC), 28692 Madrid, Spain; flopez@ucjc.edu

[4] Neuropsychopharmacology Unit, "Hospital 12 de Octubre" Research Institute, 28041 Madrid, Spain

[5] Laboratory of Medicinal Chemistry, Institute of Organic Chemistry (CSIC), Juan de la Cierva 3, 28006-Madrid, Spain; jlmarco@iqog.csic.es

[6] Instituto de Investigación en Neuroquímica, Universidad Complutense de Madrid, Ciudad Universitaria, 28040 Madrid, Spain

* Correspondence: mjoset@ucm.es; Tel.: +34-1-394-1788

† Current address: Max Planck Institute for Biology of Ageing, Joseph-Stelzmann-Strasse 9b, 50931, Cologne, Germany

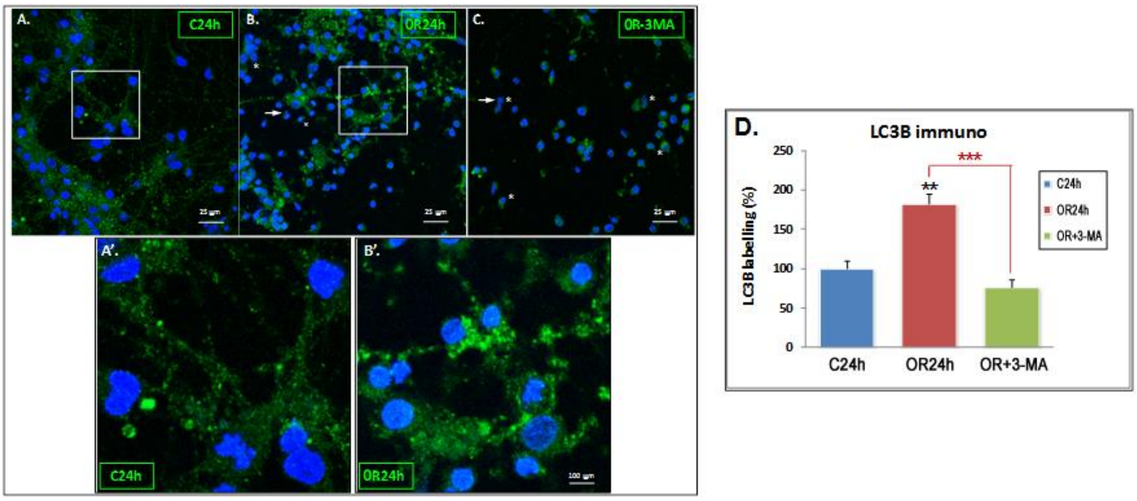

Figure S1. Effect of 3-MA on LC3B lipidation in cultured cortical neuron under OGD and OGDR conditions. **(A–C)** Confocal microscopy analysis of immunofluorescence staining of LC3B in cortical neurons under OGD and OGDR conditions. (OR). Cortical neurons were subjected to 3h OGD and 24 h oxygen and glucose resupply (OR) in the absence or presence of 5 mM 3-MA. Cells were fixed and immunocytochemistry was performed with an LC3B antibody and DAPI to stain the nucleus, as described in Material and Methods. Merge images correspond to control (**A**), OR (**B**) and OR+3-MA (**C**). Note that in the OR condition (**B**) increases the neuronal cell death and the labelling with LC3B in the surviving cells is higher than in control cells. The treatment with 3-MA (**C**) increases the number of apoptotic cells, as shown by the increase of nucleus fragmentation (*) and presence of apoptotic bodies (arrows). Scale bar: (**A–C**) 25 μm. **A′** and **B′** merge images correspond to magnifications of **A** and **B** and indicate the presence

of bigger dots of LC3B labelling in neurones under OR conditions, corresponding to autophagosome formation. Scale bar: (**A′**,**B′**) 100 µm. (**D**) Quantification of confocal microscopy images of immunofluorescence staining. Quantification was performed by counting of double labelled cells with respect to the total number of cells in the field as determined by DAPI staining. Data are expressed as mean ± SEM. Statistical analysis was done by Student *t*-test. 30 ± 15 cells per image were analyzed; $n = 8$ images from 2 different cultures, per each condition.

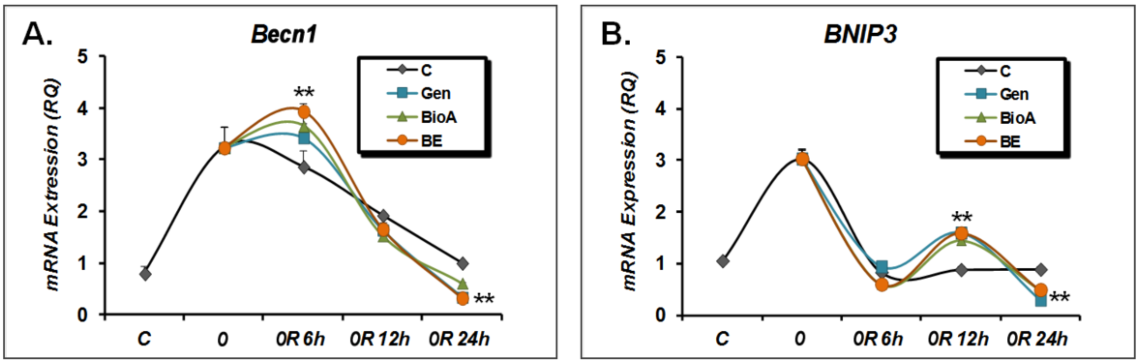

Figure S2. Effects of Phytoestrogens on Beclin 1 (Becn1) (**A**) and BNIP3 (**B**) mRNA expression in cultured cortical neuron under OGD and OGDR conditions. RT-qPCRs were performed in extracts of cortical neurons exposed to 3 h OGD (O) and 24 h oxygen and glucose resupply (OR) in the absence or presence of 1 µM genistein (Gen), biochanina (BioA) and 17-β-estradiol (BE) at indicated times during OGDR. Data are means ± SEM of three experiments, each one performed in triplicate in different cultures. Statistics compare the effect of phytoestrogens over the OR condition alone at the indicated times. (**) $p < 0.01$ (one-way ANOVA test followed by Holm−Sidak's post-hoc test).
